# Potential for Paramedic roles in Irish General Practice: A qualitative study of stakeholder’s perspectives

**DOI:** 10.12688/hrbopenres.13545.2

**Published:** 2022-08-12

**Authors:** Tomás Barry, Alan Batt, Gina Agarwal, Matthew Booker, Mary Casey, Geoff McCombe

**Affiliations:** 1 School of Medicine, University College Dublin, Dublin, Ireland; 2 Paramedic Programs, Fanshawe College, London, Ontario, Canada; 3 Departments of Family Medicine and Health Research Methods, Evidence and Impact, McMaster University, Hamilton, Ontario, Canada; 4 Centre for Academic Primary Care (CAPC), Population Health Sciences, Bristol Medical School, University of Bristol, Bristol, UK; 5 UCD School of Nursing, Midwifery and Health Systems, University College Dublin, Dublin, Ireland

**Keywords:** General Practice, Paramedicine, Qualitative Research

## Abstract

**Background**: Irish health policy emphasises the role of Primary Care and General Practice however, there is a growing shortage of General Practitioners (GPs) in Ireland. Paramedics have traditionally focused on emergency care in the community. More recently Paramedics have taken on roles in General Practice in international jurisdictions, but not yet in Ireland. This study aimed to explore key stakeholder perceptions of ‘the potential for Paramedic roles in Irish General Practice’.

**Methods**: We conducted an exploratory, qualitative stakeholder consultation study incorporating in-depth semi structured telephone interviews followed by thematic analysis. Interviews were conducted with a total of eighteen participants that included six senior Paramedics (Advanced Paramedics), seven General Practitioners (GPs), three Practice Nurses and two Practice Managers.

**Results**: Participants in this study expressed polarised views on the potential for Paramedic roles in Irish General Practice. Paramedics were enthusiastic, highlighting opportunity for professional development and favourable working conditions. GPs, Practice Nurses and Managers were more circumspect and had concerns that Paramedic scope and skillset was not currently aligned to General Practice care. GPs, Practice Nurses and Managers emphasised a greater role for expanded General Practice Nursing. There were varied perceptions on what the potential role of a Paramedic in General Practice might entail, but consensus that Government support would be required to facilitate any potential developments.

**Conclusions**: The findings of this research can inform future development of novel roles in Irish General Practice and suggests that there is appetite from within the Paramedic profession to pursue such roles. A pilot demonstration project, grounded in an action research framework could address data gaps and potential concerns. Any future developments should occur in tandem with and with due consideration for the expansion of General Practice Nursing in Ireland.

## Introduction

### Policy context

‘Sláintecare’, Irelands ten-year health strategy outlines a high-level policy roadmap for reform by reorienting the Irish health system towards integrated primary and community care ^1^. Currently, primary care services in Ireland are under increasing workforce pressure amidst a growing shortage of general practitioners (GPs) ^2, 3^. Responses such as the further development of primary care teams, the expansion of General Practice Nursing and the potential for novel roles within General Practice, have been described ^4– 6^. The Sláintecare report also highlights significant possibilities for extending the roles of Paramedics alongside other health care professionals to provide services in the community ^7^. Paramedics already actively participate in routine General Practice care in some jurisdictions such as the United Kingdom and Canada, but not yet in Ireland ^8^.

### General Practice in Ireland

Ireland’s population in excess of five million, has in the region of 4,000 GPs who provide primary care to patients and communities across the lifespan ^9, 10^. General Practice in Ireland is structured around a small business model where approximately 40% of the population qualify for free GP care contracted by the Irish State, with the remainder paying privately ^11^. Approximately 21 million GP consultations and seven million Practice Nurse consultations occur annually ^12^. More than 14% of Irish GPs are 65 years or older, with females representing a growing demographic now accounting for 42% of the workforce ^13^. Prior to the COVID-19 Pandemic, on average Irish GPs completed more than 25 face to face consultations over a ten-hour working day with 36% of overall time spent on administrative activities ^14^. Ninety percent of GPs in Ireland participate in out-of-hours care ^13^. Although GP numbers increased by 20% between 2005 to 2015, there remains significant concern (in the context of population increases and a shift toward care in the community) regarding a growing shortage of GPs, which is expected to exceed 1,000 by 2025 ^3, 15^. Significant initiatives have been launched to increase capacity in GP training, however it remains unclear whether this alone can address future demand for services ^16^.

### Paramedicine in Ireland

In Ireland, statutory emergency medical services (EMS) are provided by the Health Services Executive via the National Ambulance Service and Dublin Fire Brigade ^17^. These services respond to approximately 320,000 emergency ambulance calls annually ^18^. The scope of practice of EMS practitioners is determined by an independent statutory agency, the Pre-Hospital Emergency Care Council (PHECC) who publish clinical practice guidelines and maintain a practitioner register ^19^. Statutory EMS care is provided by PHECC registered Paramedic and Advanced Paramedic practitioners. As of July 2021, there were 2426 PHECC registered Paramedics and 700 Advanced Paramedics in Ireland ^20^. Paramedics have traditionally qualified via a two-year university diploma, and with two years additional post-registration experience can apply to undertake Advanced Paramedic training via a two-year university graduate diploma. While entry level Bachelor of Science degree qualifications have been introduced, they are not yet a requirement for professional registration. Until recently the role of Paramedics and Advanced Paramedics in Ireland has been exclusively focused on emergency care, however a recent pilot project has explored the feasibility of ‘Community Paramedicine’ where Paramedics operate in expanded primary care roles within the ambulance service ^21^.

### Paramedics in General Practice: International perspectives

The ‘Community Paramedicine’ development in Ireland mirrors international developments in paramedicine. A number of recent reviews have detailed the evolving role of paramedics in the delivery of primary care through collaborative partnerships with general practitioners, practice nurses and multidisciplinary teams, aimed at providing care in the community closer to home ^21– 23^. This is particularly true where paramedics provide access to primary healthcare in settings where it would otherwise not be available or delayed, such as in rural and isolated communities, and with populations that have historically faced barriers in accessing healthcare, such as those experiencing homelessness ^21, 24^. Paramedics have already undertaken roles in primary care assessment and onward referral, health promotion, vaccination, chronic disease monitoring and management, palliative care and urgent primary care ^23^. Community paramedicine programmes can result in a net reduction in acute healthcare utilization for enrolled patients, appear to be economically viable for the health service and result in positive patient outcomes with high patient satisfaction with care ^23^. However, the international literature demonstrates the need for a greater understanding in the areas of education, scope, role, and governance and supervision of paramedics when working in primary care settings. Without first exploring these issues paramedic roles in primary care may not realise their full potential.

### Aim

Given the health policy context in Ireland, a growing shortage of GPs and the development of Community Paramedicine, we aimed to explore perceptions of ‘the potential for Paramedic roles in Irish General Practice’ via a qualitative stakeholder consultation.

## Methods

We undertook an exploratory, qualitative stakeholder consultation study incorporating in-depth semi structured telephone interviews followed by thematic analysis. Our methodological approach was informed by O’Brien et al.’s standards for reporting qualitative research ^25^ and by the analytical approach outlined by Braun & Clarke 2008 ^26^. The type of qualitative approach employed was phenomenological; we aimed to understand participants view of their surrounding world, and how this informed their perceptions on the future potential for Paramedic working in Irish General Practice ^27^. Research design was informed by advice provided by the Health Research Board, Primary Care Clinical Trails Network Ireland, Patient and Public Involvement group.

### Ethical approval

This study received research ethics approval on the 14 ^th^ of July 2020 from University College Dublin, Human Research Ethics Committee (LS-20-35-Barry).

### Researchers’ positionality

Our research team incorporated professional perspectives from General Practice, Nursing, Paramedicine and Primary Care research. TB and GMC acted as lead researchers. Of note TB is an Academic GP who has research, education and clinical interests in emergency medical services including community paramedicine. TB is a member of the Irish Pre-Hospital Emergency Care Council and has a lead role in the development of national regulatory frameworks for community paramedicine. GMC is a research scientist with significant prior experience of qualitative research but no direct experience of community paramedicine prior to this research project.

### Participants

Interviews were conducted with a total of eighteen participants ( Table 1) that included six senior Paramedics (Advanced Paramedics), seven General Practitioners (GPs), three Practice Nurses and two Practice Managers.

**Table 1.** Participants.

Participant ID	Discipline	Sex
Participant 1	Practice Manager	Female
Participant 2	Paramedic (Advanced Paramedic)	Male
Participant 3	Paramedic (Advanced Paramedic)	Female
Participant 4	Paramedic (Advanced Paramedic)	Male
Participant 5	General Practitioner	Male
Participant 6	Paramedic (Advanced Paramedic)	Male
Participant 7	Paramedic (Advanced Paramedic)	Female
Participant 8	General Practitioner	Female
Participant 9	General Practitioner	Male
Participant 10	General Practitioner	Male
Participant 11	General Practitioner	Female
Participant 12	Paramedic (Advanced Paramedic)	Male
Participant 13	General Practitioner	Male
Participant 14	General Practitioner	Female
Participant 15	Practice Nurse	Female
Participant 16	Practice Nurse	Female
Participant 17	Practice Nurse	Female
Participant 18	Practice Manager	Female

### Recruitment

As this exploratory study was one of the first to consider the potential for Paramedic roles in Irish General Practice, we hypothesised that key stakeholders were patients, members of the Paramedic profession, General Practitioners, Practice Nurses and Practice Managers. In this study we focused our data collection on professional groups only. Planned follow-on work will consult directly with patients as a distinct group.

We employed a purposive approach to sampling. At the outset we targeted a total sample of between 16–20 participants with approximate thirds representing Paramedics, GPs, and Practice Nurses/Managers (as a combined group). We based this sample size target on our previous experience of conducting qualitative research and the overall aim of achieving a thorough understanding of the phenomenon under study ^28– 30^. We considered GPs and Paramedics to be core initial stakeholders with Practice Nurses and Managers also representing important stakeholders. Our sampling frame sought to achieve maximum-variation sampling considering gender and location of practice ^30^. Recruitment occurred in an incremental fashion, and continued until we considered that an appropriate sample size had been achieved, one that could facilitate a thorough understanding of the phenomenon under study. Over the recruitment process a single email invitation was sent to a total of twenty GPs, eleven Paramedics and five Practice Managers. GPs, Paramedics and Practice Managers were all drawn from the University College Dublin (UCD) professional network. UCD is Irelands largest medical school and has provided academic training programs for Paramedics and Advanced Paramedics since 2005 ^31^. The University actively engages a large pool of adjunct clinical faculty that supports undergraduate medical General Practice (approximately 200 GPs) and Paramedical training (approximately 60 Advanced Paramedics). The University also has engagement with GP Practice Managers around undergraduate student placements. Practice nurses were recruited via the professional networks of Professional Development Coordinators for Practice Nursing who disseminated an invitation to participate on behalf of the research team.

### Data collection

One researcher (GMC) conducted all interviews, which were via telephone and took place between January and October 2021. Prior to conducting the interviews TB & GMC formally planned the interview process. A semi-structured interview format was adopted to allow flexibility of response, and follow up ‘unplanned’ questions via a participant led exploration of topics ^27^. Our approach involved the development of a topic guide that facilitated outline structure but did not mandate rigid adherence ^32^. This guide was informed by a prior scoping review ^8^. The guide included five domains: general awareness, acceptability, feasibility, potential and challenges. Each domain involved standard initial open-ended prompt questions and flexible follow-on questions based on the interviewee’s initial responses. A copy of the topic guide can be found in the *Extended data* ^33^. Informed written consent was obtained before commencing each interview. Interviews ranged between ten- and twenty-four-minutes duration, (average seventeen minutes). The initial interview acted as a pilot. Brief field notes were kept throughout the interview process. All interviews were audio recorded using Skype and later professionally transcribed. TB then checked against the recordings for accuracy, made minor corrections as necessary, and removed any information that was considered to identify participants. Transcripts were stored electronically as password protected files.

### Data analysis

Data were analysed using the thematic analysis (TA) framework outlined by Braun and Clarke that includes six phases: ‘Data familiarisation’, ‘Generating initial codes’, ‘Searching for themes’, ‘Reviewing themes’, ‘Defining and naming themes’ and ‘Producing a report ^26, 34^. This framework was selected based on the research teams prior experience of employing the methodology, its accessibility as a qualitative research approach and its flexibility in terms of theoretical framework ^32^. Initial coding and all further phases of TA were supported by NVIVO 12 for Windows. TB and GMC acted as co-researchers during the analytic process to ensure credibility. TB coded all transcripts while GMC coded a sample of four transcripts independently. Both TB and GMC reviewed all candidate and final worked out themes thus supporting a reflexive process throughout the data analysis ^34^. The analysis ultimately produced a structure of themes relating to the key domains that reflect the focus of our study.

## Results

### Overall structure of themes

**Figure 1.**
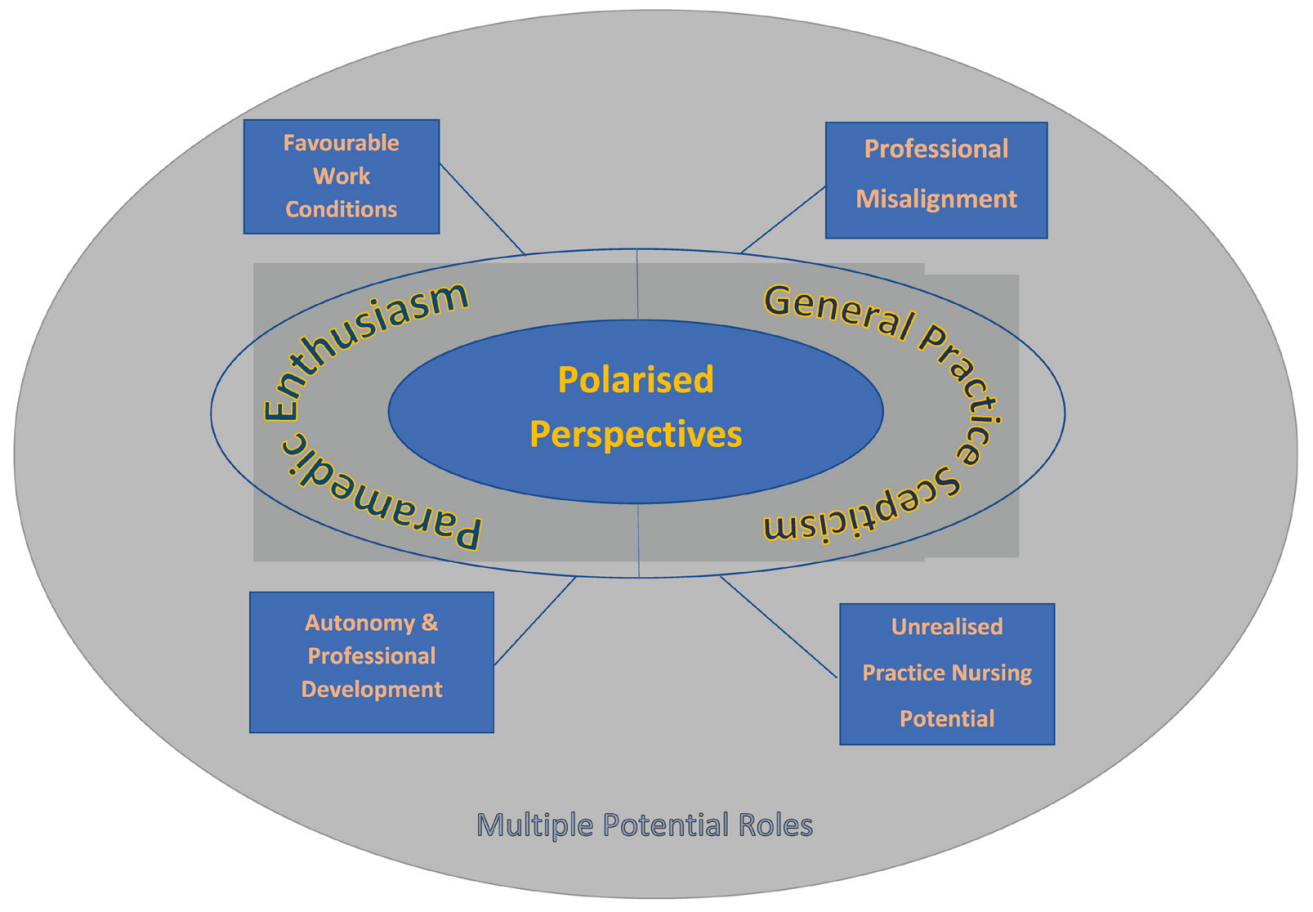
Overall structure of themes.

Comprehensive data analysis generated a structure of themes as illustrated in Figure 1, and described below. A central organising theme was that of ‘polarised perspectives’ where Paramedic participants expressed enthusiasm for the concept of Paramedics working in General Practice while the General Practice community were by in large more sceptical. This central theme was driven by four distinct sub-themes. In terms of Paramedic enthusiasm these subthemes related to a perception that novel roles would offer the opportunity for increased autonomy and professional development and in addition have more favorable working conditions. In contrast, General Practice scepticism was driven by a perceived misalignment between the professional nature of Paramedicine and General Practice. In addition, there was a definite sense that addressing unrealised Practice Nursing potential was a higher immediate priority for General Practice.

Beyond this central thematic structure, a background sub-theme represented a function of the large variety of potential future Paramedic roles within General Practice suggested across all participant groups. Suggested roles varied across in and out-of-hours care, chronic and acute disease management as well as in terms of levels of responsibility and complexity. There was however consensus that formal government support would be required to allow the development of any such roles.

### Polarised Perspectives: Paramedic Enthusiasm & General Practice Scepticism

The senior Paramedics interviewed were uniformly supportive of the concept of Paramedics working in General Practice.

‘ *I would very much see a role for Paramedics in General Practice’. - Participant 2 Paramedic.*

However, most GP’s, Practice Nurses & Managers were more circumspect.

*‘I suppose I would have been a bit surprised in one way. I wouldn’t have thought there would have been a role for Paramedics in General Practice as such’. - Participant 11 GP.*
‘ *I just don’t see, it’s an extra person put in there when we don’t need them, basically, because we’re all able to do what they’re going to be doing.’- Participant 17 Practice Nurse.*
*‘I don’t see it is quite feasible or to an advantage to GP practices.’ - Participant 18 Practice Manager.*

### Paramedic Enthusiasm: Autonomy & Professional Development 

Paramedics interviewed highlighted the potential increasingly autonomous nature of new roles.

*‘My thoughts on what Paramedics could bring to General Practice, is probably an element of autonomous practice, complementing GPs’. - Participant 4 Paramedic.*

From the Paramedic perspective such roles in General Practice were particularly attractive in terms of opportunities for professional development.

*‘If you're involved in a role like that in a GP surgery, you know, I suppose you are allowed to grow and to develop’ - Participant 3 Paramedic.*

In terms of alignment with a broadening scope of practice, Paramedics did feel that these novel roles would be appropriate for senior, experienced Paramedics.

‘ *I would have to qualify this by saying I am not talking about every licensed Paramedic in Ireland. I am talking about a senior experienced “specialised Paramedic” who now works in that role’. - Participant 2 Paramedic.*

Paramedics also highlighted the need for specific training and for mentorship in preparation for these proposed new roles. They highlighted that the nature of this preparation should be tailored to what the role will incorporate.

*‘Yeah, I think it certainly would be advisable to have a training programme. I mean I think it really depends on the scope of practice within the GP environment.’ - Participant 12 Paramedic.*

One specific area highlighted as requiring further training was mental health presentations. This was described as a significant issue relevant to both novel roles in General Practice and relevant in terms of day-to-day emergency work.

*‘I have been an outspoken advocate for further training in a lot of these areas for a lot of years, for the simple reason our mental health patient training is bordering on non-existent. Yet we deal with a disproportionately large amount of mental health patients in our day-to-day work’. - Participant 2 Paramedic.*

This issue was also highlighted by one GP participant.

*‘Their mental health training – I get the impression it is not fantastic and they kind of learn on the job. They learn empathy and stuff like that, on the job, through exposure, but whether you would have them accessing people, I don’t know. I suppose with a bit of additional training in that area would help.’ - Participant 5 GP.*

### Paramedic Enthusiasm: Favourable Work Conditions

Paramedics described how novel roles could be attractive at the later stages of their careers in terms of more sociable hours, better work conditions and salary.

*‘For the individuals who are more senior they would see an attraction to it, In that a) it takes you off nights and sort of shift-work to a great extent, and you're extending your skillset and your knowledge base.’ - Participant 6 Paramedic.*

Paramedics noted that experienced colleagues might also be less motivated by the excitement or pace of emergency care and more willing to work in the less acute environment of General Practice.

*‘It would certainly be a role that Paramedics that have been in their job a long time or maybe that are burnt out from being on the road so much or need a change and want to be involved in a community, it would be certainly a route for them to go.’ - Participant 7 Paramedic.*

In parallel though there was some recognition that the small business context of General Practice could also imply less stable employment arrangements, that may not be attractive to those coming from public service employment.

*‘Most Paramedics in the country are kind of working within statutory organisations where, you know they have a career for as long as they want it, you know. Just moving into the private sector, would probably be a little bit daunting for some individuals.’ - Participant 4 Paramedic.*

### General Practice Scepticism: Professional Misalignment

GPs, Practice Managers and Nurses highlighted a discordance between the traditional emergency focus of Paramedic training and the nature of General Practice. Although they acknowledged some overlap in terms of Paramedic and GP practice emergencies, General Practice was considered a distinct domain unlikely to be reflected in Paramedic training.

*‘I always think of a Paramedic as minor trauma, major trauma, acute cardiac care. They are absolutely fantastic. I step aside when they arrive on the scene, but I would only see those two or three times a year max in my own practice.’ - Participant 8 GP.*

Key issues relevant to General Practice were highlighted including continuity, complexity, and reassurance. These issues were not considered to be core to Paramedic training or experience.

*‘I suppose I have some doubts about it, because I think the way Paramedics and GPs are trained is very different and the type of presentations that we deal with on a day-to-day basis. We see a lot of minor illness. We do a lot of reassurance. We deal with complexity. We look after people in the longer term, so there is continuity of care, and very often consultations involve two or three problems. We manage chronic disease maybe in an acute illness. Whereas I think Paramedics and maybe I am wrong about this, they make a decision about whether somebody needs acute care, whether they need hospitalisation and I suppose they deal with a lot of trauma and acute serious illness.’ - Participant 14 GP.*

Some expressed significant concern that Paramedics would not be able to adapt their practice to the primary care context.

*‘So, my concern would be the versatility of the Advanced Paramedics would need to be explored a bit more before they would deal with the totality of taking on some of the burden of primary care’. - Participant 13 GP.*

Others highlighted the significant differences in duration and depth between Paramedic and GP training.

*‘But I am not necessarily convinced that a Paramedic who is going to have not the same training as a GP, can out rule maybe serious things all the time.’ - Participant 14 GP.*

Some participants believed Paramedic training and skills would be more appropriately deployed in environments other than General Practice.

*‘I think it would be a waste of their specialist training, because like I said, the Paramedic has such highly specialist, acute, emergency, that type of training.’ - Participant 16 Practice Nurse.*

Despite the assertion that the General Practice context was not a natural fit for Paramedics, the general sense was that patients were likely be open to Paramedics providing General Practice care.

*‘I think patients, as long as you’ve got a professional, well-educated healthcare professional … as long as the person got the care they needed, appropriate care, appropriate time, appropriate all of that. I don’t see patients having an issue. I think they trust that if we, as a healthcare professional, whoever it is, looks after them properly, that they tend to trust our services.’ – Participant 16 Practice Nurse*.

In some contrast to the General Practice concern around professional misalignment, Paramedics did highlight very significant exposure to low acuity conditions during their experience of working in the EMS system.

*‘I mean, certainly in my own job like, I see loads of people that present to the ED that really you know, a GP practice or a good kind of primary care clinic would be able to address their needs quite well.’ -Participant 4 Paramedic.*

In addition, they highlighted their generalist nature, having experience of a breadth of undifferentiated presentations across all age groups.

*‘I think Paramedics are in the prime position to do that. They’ve been out on ambulances, they're going to houses all the time, seeing a variety of illnesses and situations and in general, nothing really surprises them.’ - Participant 7 Paramedic.*

### General Practice Scepticism: Unrealised Practice Nursing Potential

Throughout the interviews GPs, Practice Nurses and Practice Managers highlighted the current key role and future potential of ‘Practice Nursing’. A recurrent issue raised was whether the addition of Paramedics to General Practice could bring relevant additional skills and capacity over and above what could be accessed via further resourcing and developing Practice Nursing in Ireland.

*‘My immediate gut feeling was what would they add to the team that I already have? I am not seeing any huge advantage. And what I mean by that is, we have two very experienced, well-trained nurses.’ - Participant 8 GP.*
*‘I don’t see why bringing in (Paramedics) because there’s a shortage of GPs, why would you choose a Paramedic over a nurse?’ - Participant 17 Practice Nurse.*

One Practice Manager observed that Paramedics would need significant additional training to work in General Practice whereas nursing training was viewed as more applicable to the General Practice setting.

*‘I would also be a bit concerned initially that they would need a lot of training and adapting if it is a new sort of scope of practice, that we would need to train them up for several months and to figure out where to put them. Whereas a nurse again would probably hit the ground running a lot quicker in terms of running a General Practice.’ -Participant 1 Practice Manager.*

The potential for novel advanced nurse practice roles, with more responsibilities in the context of a GP shortage were highlighted. This was perceived as a more logical way to solve a GP staffing crisis.

*‘I mean, why, because already we have Advanced Nurse Practitioners within General Practice, and an Advanced Paramedic, what is their skill set? Advanced Nurse Practitioners and all Practice Nurses manage chronic diseases, manage diabetes, manage all that type of thing, so are they going to be educated to that level to be able to do that?’ - Participant 17 Practice Nurse.*

One GP framed the issue in terms of the allocation of scare resources and opportunity cost. Their perspective was that as nursing was already established in General Practice, resources would be better deployed in enhancing the nursing role rather than developing a new role.

*‘For me, I would see that as more advantageous, simply because we have such a close working relationship with our Practice Nurses. I would be concerned if I saw investments going into two streams where money is obviously precious and I would be saying, ‘Well, why are they upskilling this group as well as this other group, when we don’t need to be upskilling both?’ - Participant 9 GP.*

Another GP highlighted the risk that Paramedic working in General Practice could result in a workforce shortage in front line emergency ambulance services.

*‘A little concern is there aren’t a huge number of Advanced Paramedics in the community at the moment, within the employment of the HSE. You worry about taking away a scarce but valuable commodity and putting it into somewhere else that you may be able to find an alternative option.’ – Participant 13 GP.*

Several participants from the General Practice setting highlighted the alignment of existing GP funding with Practice Nursing and revenue streams.

*‘I would imagine their (Paramedics) expenses would be about the same as a Practice Nurse. On that basis I can see a much better integration as additional Practice Nurse, being able to do the full range of chronic disease management and things that are actually lucrative at the moment.’ - Participant 1 Practice Manager.*

Although most GPs, Practice Managers and Practice Nurses were sceptical as to what additional benefit Paramedics would bring to General Practice above and beyond a Practice Nurse, this was not universal. One GP believed Paramedics would be a good fit for General Practice in terms of their communication skills, attitude and ability to integrate.

*‘They (Paramedics) are well suited to the General Practice environment. Any of them that I have met, and I have probably met about half a dozen at this stage, would slot in, usually in General Practice, just primarily in terms of their attitude. They are nearly universally pleasant to be around, always courteous and very professional. They are very knowledgeable’. - Participant 5 GP.*

Other participants highlighted the reality of a shortage of Practice Nurses in addition to GPs. These participants acknowledged that there may not be sufficient GP or nursing supply to manage work demands in General Practice into the future.

*‘Because of the sheer pressure that is out there on General Practice at the moment and bringing in chronic disease management, I don’t think that you will find many nurses probably willing to upscale to that because they understand the additional pressures and work commitments that goes with that’. - Participant 18 Practice Manager.*

One Practice Nurse specifically linked the nursing supply issue to the terms and conditions of employment in General Practice, which were perceived as inferior to the hospital environment.

*‘So, I would perceive that there is currently a shortage of Practice Nurses, and I don’t see things improving in the near future because I think the Department of Health would be very reluctant to make them state employees…I think they’re paying about 24, maybe 25 euros an hour, which is quite a bit of a pay drop for a lot of nurses. ... Most practice employees do not have sick leave. They might have a fortnight if they’re lucky...so you don’t get things like maternity benefits, you don’t get your full salary if you go on maternity benefit.’ - Participant 15 Practice Nurse.*

Other Practice Nurses expressed concern that the addition of Paramedics to General Practice could undermine development and resourcing of the Practice Nursing role. There was a concern that by pursuing Paramedic involvement in General Practice, public resources would be channelled away from the development of Practice Nursing.

*‘I’m interested to hear that Paramedic is a totally different field of healthcare professional, that there’s a focus on putting a course there for them, though they’re not even part of that setup at all, and the General Practice Nurses are completely forgotten about.’ - Participant 16 Practice Nurse.*

Another Practice Nurse highlighted the difficulty with potential employment arrangements in General Practice and a perception that Paramedics working in General Practice would continue to be directly employed by the public health service, and thus have access to benefits not currently enjoyed by Practice Nurses.

*‘If they are employed by the HSE (public health service) they will have a huge amount of backing and education and the whole lot; whereas, because Practice Nurses are employed by GPs, any education is at the discretion of the GP to fund it or send them or whatever, so what should be happening is, the nurses and advanced nurses should be actually employed by the HSE and get...and upskill them that way.’ - Participant 17 Practice Nurse.*

### Varied perceptions of Multiple Potential Roles for Paramedics Working in General Practice

Across the participants interviewed there were varying perceptions in terms of the potential scope of practice of a Paramedic working in General Practice and the roles that one might fulfil.

Some participants highlighted the potential to take on domiciliary care thus freeing up GP time.

*‘I think the role would arguably lend itself to a community Paramedic, whereas you are potentially going out on behalf of the GP. But I am acutely aware of how much pressure GPs are under at the moment given the current environment, and maybe filling that gap where they don’t have time to do home visits and then liaising directly back with the GP.’- Participant 2 Paramedic.*

Others envisaged the role as revolving around acute problems or acute presentations of chronic illness.

*‘I really think that they are more for the assessment of the new problem or the deterioration of chronic problems.’- Participant 5 GP.*

One Practice Manager took a pragmatic view. If a Paramedic working as an advanced practitioner could demonstrate the capacity to cover for periods of leave or GP shortages this would be considered an attractive proposition in the current workforce shortage climate.

*‘I think it would be interesting to see how far they would be upskilled and how close their skill set would be to a GPs. As an advanced practitioner, you know, I think that is definitely something that our surgery would look at, particularly it if you have the likes of holidays. You have a lot of GPs away on leave. You could certainly look at bringing in the likes of that as opposed to if you couldn’t get a locum. Yeah, I would explore that.’ - Participant 18 Practice Manager.*

At the other end of the spectrum some participants envisaged that Paramedics working in General Practice could free up nursing time by taking on some of the more straightforward or procedural duties currently undertaken by Practice Nurses.

*‘I think a lot of nurses would be happy to take on more highly skilled roles if they weren’t so busy doing all the things like the bloods and the routine stuff. That’s my opinion on it anyway. Yes, I definitely would be a big fan.’ - Participant 15 Practice Nurse.*

Some participants did highlight that the concept might have specific relevance in out of hours care and in rural areas where the GP workforce shortage is particularly acute.

*‘In fact, the Paramedics have taken over GP roles now in house calls and emergency calls in that they do the assessment. They obviously assess the patient’s condition now, so they make decisions whether they need to go to hospital or not and we have advocated this role in the co-ops and out-of-hours setting where an ambulance is called directly rather than the GP.’ - Participant 11 GP.*

Independent of the specifics of scope and role, participants uniformly highlighted the need for government support and funding to facilitate Paramedic working in General Practice. In particular, the small business nature of General Practice was highlighted as a potential challenge.

*‘You will have to provide them with their own room and working environment. And the other thing, being a GP, you own the business, so you have a vested interest in it. So, it is a slightly different environment that they would be coming from… They would have to transform over to a slightly different model of practice, whereby income generation is key, because you have to pay your own way. So, that is the financial consideration.’ - Participant 13 GP.*

In addition to Paramedic renumeration, participants again highlighted that support would be needed in terms of training and also regulation.

*‘It makes sense as a health service to say we could facilitate progression within the General Practice setting, to alleviate some of the shortcomings due to sheer numbers. And in that case, I think, certainly, the pre-hospital care council or the HSE (national health service) or some governmental organisation can take over the training and qualification, and credential setting for the practitioners. I think if you rely on people to do it privately, it is going to be a lot more difficult, but if it is an avenue that is open to people and that is being funded by the State, I think it is going to be a lot easier to get people into the system.’ - Participant 12 Paramedic.*

## Discussion

### Summary

Participants in this study expressed polarised views as to the overarching potential for Paramedic roles in Irish General Practice. The senior Paramedics (Advanced Paramedics) interviewed were positive about the concept citing opportunity for professional development and favourable working conditions. They detailed significant experience of lower acuity presentations via their existing day to day roles but considered additional training for novel General Practice roles a necessity. Participants from the General Practice community (GPs, Practice Nurses and Practice Managers) were more circumspect and had concerns that Paramedic’s core training and scope of practice was not aligned to most General Practice care.

Although not a specific element of our research question, participants from general practice (GPs, Practice Nurses and Practice Managers) noted the issue of the current role and future potential of General Practice Nursing as a priority. For some, consideration of Paramedic roles in General Practice was considered a distraction from what was a greater priority. There was a sense that the potential of General Practice Nursing had not yet been realised and that systemic barriers existed that must be addressed in the first instance. In addition, participants discussed the employment model of Practice Nursing within a small business framework, and with limited state support for further professional development.

Participants had varied perceptions of the nature of potential roles for Paramedics in General Practice that included elements of domiciliary care, same day acute presentations and chronic disease management. For some, the role could involve autonomous decision-making, bridging that of the Practice Nurse and GP and for others the role would involve concrete procedural tasks that could free up nursing time. Out-of-hour’s care and rural practice were considered to be specific areas of current workforce supply-demand mismatch and thus were seen as most relevant to potential Paramedic roles. There was uniform agreement across participants that state support would be necessary in terms of educational and service delivery elements of any future model.

### Comparison with existing literature

In keeping with the theme of ‘varied perceptions of the potential role’, the wider literature also notes significant real-world variation in terms of Paramedics working in General Practice roles, often reflecting differing training and support mechanisms as well as local circumstances ^8, 35^. Booker and Voss have suggested three overarching models for Paramedic working in General Practice: Paramedics provide routine patient care thus freeing GP time to manage more complex issues; Paramedics create additional capacity and thus allow general practice to manage urgent problems in a timely manner; Paramedics provide timelier and longer consultations and thus have the potential to improve patient satisfaction and clinical outcomes ^36^. Participants in our study noted that there was potential for paramedics to provide additional general practice capacity in Ireland across these three models.

The degree of scepticism toward the concept of Paramedics working in General Practice expressed by the General Practice community in this study does not appear to be reflected widely in the literature ^8^. A degree of scepticism is however understandable in the context of a current relatively limited evidence base considering clinical and cost effectiveness as well as safety ^8, 22, 37– 39^. That said the scepticism identified in this current study does appear to contrast with a largely positive view of Paramedics working in General Practice held elsewhere, one that frequently relates to a perceived association with reduction in GP workload ^22, 37^. The results of our study suggest that the Irish General Practice community may be unaware of the evidence and thus wider possibilities of ‘Community Paramedicine’ in both chronic disease management and in addressing gaps in primary care provision. For instance, research from Canada demonstrated that Community Paramedic programs can act as primary care extenders and met the needs of vulnerable populations ^24^. In Ontario, a Paramedic-led community-based program for older adults living in subsidized housing was found to be cost effective, lowered blood pressure and was associated with improved functional independence ^40, 41^. Interestingly, a recent additional study from Ireland that focused on rural General Practitioners, suggests that this specific group may be significantly more enthusiastic about the potential for paramedic involvement in the near term ^42^.

Though not the focus of our study our research does highlight the importance and potential of the General Practice Nursing role in Ireland. There appears to be a dearth of scientific literature considering this domain despite the observation that approximately one third of all clinicians working in General Practice in Ireland are Practice Nurses ^43^. Previous research suggested that the role of the Practice Nurse in Ireland centred on immunisation, direct clinical care and elements of chronic disease management ^44^. Recent research involving a survey of Practice Nurses and GPs in one county found that the large majority considered an expanded role for practice nurses to be a high priority ^6^. Further research is ongoing and aims to quantify the provision of quality Nursing care in Irish General Practice ^45^.

Participants from General Practice in this study raised concerns regarding the potential for role conflict and competition for resourcing when considering Nursing and Paramedicine in the General Practice Setting. The wider literature does highlight potential antagonism towards new Paramedic roles in Primary Care but suggests that this can be resolved through exposure, familiarity, and the clarification of scope of practice from the outset ^8, 46, 47^. The importance of full integration with the primary care workforce has been identified as a key feature of successful models of Paramedic working in primary care ^21^. The interface of GP, Practice Nurse, Paramedic and other Primary Care Staff warrants ongoing consideration. An important component of the Irish ‘Sláintecare’ strategy is the facilitation of efficient models that allow health care professionals to work to the ‘full scope of their licence’ ^48^. The results of this study suggest significant further potential for the Practice Nursing Role at least in tandem with (and perhaps before) any major drive to recruit Paramedics into General Practice.

Our previous scoping review noted a lack of literature that explored the motivation of Paramedics to transition to General Practice roles ^8^. In this study we found that a desire for further professional development as well as increased autonomy may be important motivating factors. Furthermore, Paramedics at a certain stage of their careers may perceive the work conditions in General Practice to be more favourable than in front line ambulance care. It remains to be seen whether these perceptions will in fact be realised in any model that emerges. Notably both Practice Nurses and GPs highlighted significant existing issues in terms of the employment conditions and existing professional supports relevant to Practice Nursing.

### Strengths and limitations

Our study has several strengths that support its ability to inform understanding of this topic. These include the use of published reporting guidelines to inform design from the planning stage, consultation with a patient and public advisory group, and the use of a prior scoping review to inform the development and implementation of this research. However, there are some important limitations that should be considered in interpreting our results. The interviews conducted yielded a rich understanding of participants perspectives across a range of relevant disciplines. It is however not clear to what extent these perspectives apply more generally outside of the research sample. Most participants had some affiliation or prior relationship with University College Dublin, and this may have influenced their perceptions. Ultimately the findings need to be viewed as hypothesis generating and further research is needed to establish whether the findings are generalisable to the wider population of Paramedics, GPs, Practice Nurses and Practice Managers in Ireland.

### Implications for research and practice

The data collected in this study suggests that Paramedics may be well disposed to the concept of working in General Practice however the General Practice community may be more circumspect. Further research should be conducted to ascertain whether these findings apply more generally in Ireland. If General Practice reticence is widely held this will represent a significant barrier to be overcome as any initiative is progressed. A recent pilot project has involved ‘Community Paramedics’ operating in expanded primary care roles within the Irish ambulance service [20]. These Community Paramedics spend time in designated GP practices during their training and maintain a relationship with that practice after. The small cohort of GPs (and potentially Practice Nurses and Managers) involved in this pilot will have now accrued significant exposure to the Community Paramedicine concept and are likely to have a key perspective on the future potential of Paramedic roles within core General Practice. The perspective of this group is not captured within this current research but may be critical in informing future developments. While focusing on Paramedicine our research also highlights the importance of Practice Nursing roles as a key issue for Sláintecare, General Practice and the Irish health system. It appears crucial that any novel Paramedic roles in General Practice are developed in tandem with and with due consideration for General Practice Nursing, both as currently configured and as envisaged into the future. Given these observations coupled with a current lack of evidence considering patient centred outcomes and cost, novel models involving Paramedic working in General practice should be piloted within a research framework that gathers real world evidence to inform developments ^8^.

## Data availability

### Underlying data

As this is a qualitative study the underlying data represents audio recordings and written transcripts that have been derived from the recordings. The participants have been guaranteed anonymity in the consent form, and the studies ethical approval requires that both the recordings and transcripts are not shared outside the study team. Therefore, underlying data for this study cannot be shared with other researchers. The Methods section contains detailed information to allow replication of the study. Any queries about the methodology should be directed to the corresponding author.

### Extended data

Open Science Framework: Potential for Paramedic Roles in Irish General Practice: A Qualitative Study of Stakeholder’s Perspectives. https://doi.org/10.17605/OSF.IO/7TA35 ^33^.

This project contains the following extended data:

- Potential for Paramedic Roles in Irish General Practice: A qualitative study of stakeholder’s perspectives (topic guide)

Data are available under the terms of the Creative Commons Zero "No rights reserved" data waiver (CC0 1.0 Public domain dedication).
