# Supplementary material for: Potential for Paramedic roles in Irish General Practice: A qualitative study of stakeholder’s perspectives
Source: HRB Open Res. 2022 Aug 12;5:40. Originally published 2022 May 23. [Version 2] doi: 10.12688/hrbopenres.13545.2 (PMC9418754; doi:10.12688/hrbopenres.13545.2)
Supplement: Peer review history

# Peer review: Reviewer response for version 2

**DOI:** 10.21956/hrbopenres.14850.r32670

**Published:** 2022-08-26

**Authors:** Blandine French

**Affiliations:** 1 Academic Unit of Mental Health & Clinical Neurosciences, Faculty of Medicine & Health Sciences, University of Nottingham, Nottingham, UK

No further comments

Is the work clearly and accurately presented and does it cite the current literature?

Yes

If applicable, is the statistical analysis and its interpretation appropriate?

Partly

Are all the source data underlying the results available to ensure full reproducibility?

No source data required

Is the study design appropriate and is the work technically sound?

Yes

Are the conclusions drawn adequately supported by the results?

Yes

Are sufficient details of methods and analysis provided to allow replication by others?

Yes

Reviewer Expertise:

Primary care, neurodevelopmental disorders, thematic analyses

I confirm that I have read this submission and believe that I have an appropriate level of expertise to confirm that it is of an acceptable scientific standard.

# Peer review: Reviewer response for version 1

**DOI:** 10.21956/hrbopenres.14787.r32341

**Published:** 2022-06-23

**Authors:** Tony Foley

**Affiliations:** 1 Department of General Practice, University College Cork, Cork, Ireland

Many thanks for the opportunity to review this interesting paper exploring the important topic of relevant stakeholders' perspectives on the potential for paramedic roles in Irish General Practice. The study is clearly presented, appropriately designed, and reported in detail. 

 I have offered some comments below which I hope the authors will find constructive.

**General Practice in Ireland:**

- Consider adding more up-to-date detail regarding the ICGP response to increasing the number of GP trainees.

**Paramedics in General Practice: International perspectives:**

- You note there has been a recent detailed review of community paramedicine. I think it would be helpful to add a sentence here offering examples of the actual/specific roles undertaken by community paramedics. Have these roles been evaluated, and if so what do we know about their success?

**Data Collection:**

- Was the interview pilot-tested?
- Were field notes made?
- What were the durations of interviews?
- Were transcripts returned to participants for comment afterward?
- Was data saturation achieved?

**Paramedic Enthusiasm: Preparation tailored to the role:**

- " *In addition, they highlighted their generalist nature, having experience of a breath of undifferentiated presentations across all age groups.*" - I think that word should be ‘breadth’.

**Discussion:**

- Regarding the degree of scepticism, it is stated that there is a lack of evidence of the cost-effectiveness and clinical outcomes – but then evidence for this is offered towards the end of that paragraph. Perhaps it is more about GPs being unaware of the effectiveness or evidence-base for the role? 
- In jurisdictions where the role has evolved and become integrated into general practice, has scepticism persisted?
- In addition, I’d be interested in knowing whether paramedics’ enthusiasm for their GP role is echoed in the wider literature and whether that positivity has been maintained where the role has progressed and become integrated into general practice.
- I’m unsure about including the paragraph on the expanding role of practice nurses, as you concede this is not an integral part of your research, or indeed a research question. I wouldn’t have thought a full paragraph in this section is warranted. If it is included, perhaps there should be an analysis of the evidence in the literature on the role of nurse vs paramedic in general practices.

**Strengths and Limitations:**

- I’m unsure what you mean when stating that a strength was that the study used published guidelines.
- Unless I missed it, this is the first mention I can see of PPI involvement. Consider inserting it into the methods section too.

Again, many thanks and best wishes.

Is the work clearly and accurately presented and does it cite the current literature?

Yes

If applicable, is the statistical analysis and its interpretation appropriate?

Not applicable

Are all the source data underlying the results available to ensure full reproducibility?

No source data required

Is the study design appropriate and is the work technically sound?

Yes

Are the conclusions drawn adequately supported by the results?

Yes

Are sufficient details of methods and analysis provided to allow replication by others?

Yes

Reviewer Expertise:

General practice, primary care, older person care, dementia care

I confirm that I have read this submission and believe that I have an appropriate level of expertise to confirm that it is of an acceptable scientific standard.

# Response

**Published:** 2022-07-06

**Authors:** Tomás Barry

Thank you for your review and suggestions. Please find our responses to the issues raised below.

*General Practice in Ireland:*

*Consider adding more up-to-date detail regarding the ICGP response to increasing the number of GP trainees.*

 Thank you for this reference which we have added.

*Paramedics in General Practice: International perspectives:*

*You note there has been a recent detailed review of community paramedicine. I think it would be helpful to add a sentence here offering examples of the actual/specific roles undertaken by community paramedics. Have these roles been evaluated, and if so what do we know about their success?*

 We have added the following:

 ‘Paramedics have already undertaken roles in primary care assessment and onward referral, health promotion, vaccination, chronic disease monitoring and management, palliative care and urgent primary care.22 . Community paramedicine programmes can result in a net reduction in acute healthcare utilization for enrolled patients, appear to be economically viable for the health service and result in positive patient outcomes with high patient satisfaction with care 22.’

*Data Collection:*

*Was the interview pilot-tested? Were field notes made? What were the durations of interviews?*

 We have added – ‘Interviews ranged between ten- and twenty-four-minutes duration, (average seventeen minutes). The initial interview acted as a pilot. Brief field notes were kept throughout the interview process.’

*Were transcripts returned to participants for comment afterward?*

 No, but as mentioned in the methods they were checked against the tapes for accuracy.

*Was data saturation achieved?*

 We did not incorporate this concept as Braun & Clarke do not advocate the use of this measure in their method and suggest that it is not consistent with the values and assumptions of reflexive TA. See Virginia Braun & Victoria Clarke (2021) To saturate or not to saturate? Questioning data saturation as a useful concept for thematic analysis and sample-size rationales, Qualitative Research in Sport, Exercise and Health, 13:2, 201-216, DOI: 10.1080/2159676X.2019.1704846

 Interestingly a further recent study from Ireland that focused on rural GPs found that this group were more enthusiastic about the introduction of Paramedics to General Practice.

 Feerick, F., Connor, C.O., Hayes, P. et al. Introducing Advanced Paramedics into the rural general practice team in Ireland – general practitioners attitudes. BMC Prim. Care 23, 130 (2022). https://doi.org/10.1186/s12875-022-01740-9

 This would suggest that diverse perspectives may exist among different sub-groups of GPs. This additional publication is now cited in the discussion section.

*Paramedic Enthusiasm: Preparation tailored to the role:*

*"In addition, they highlighted their generalist nature, having experience of a breath of undifferentiated presentations across all age groups." - I think that word should be ‘breadth’.*

 Thank you – corrected as advised.

*Discussion:*

*Regarding the degree of scepticism, it is stated that there is a lack of evidence of the cost-effectiveness and clinical outcomes – but then evidence for this is offered towards the end of that paragraph. Perhaps it is more about GPs being unaware of the effectiveness or evidence-base for the role?*

 Thank you for this observation – we have qualified the evidence base as ‘relatively’ limited rather than limited given as you say there is some encouraging evidence for the concept. It is probably fair to say that the evidence base is at an early stage in its development.

 We have amended the text to read :

 ‘The results of our study suggest that the Irish General Practice community may be unaware of the evidence and thus wider possibilities of ‘Community Paramedicine’ ‘

*In jurisdictions where the role has evolved and become integrated into general practice, has scepticism persisted?*

 Anecdotally this may be the case, at least to some degree in some sectors, however existing literature does suggest a ‘largely positive view of Paramedics working in General Practice held elsewhere, one that frequently relates to a perceived association with reduction in GP workload 21, 36’

*In addition, I’d be interested in knowing whether paramedics’ enthusiasm for their GP role is echoed in the wider literature and whether that positivity has been maintained where the role has progressed and become integrated into general practice.*

 In general, the literature thus far appears to have focused more on the positivity originating from primary care. In the paper we reference our scoping review where we ‘noted a lack of literature that explored the motivation of Paramedics to transition to General Practice roles 8’

 It may be of interest to note though that many of these issues are being further explored in ongoing research work led by our-co-author Dr Booker – see https://www.uwe.ac.uk/research/centres-and-groups/chcr/research-themes/emergency-care/ready-preliminary . The outputs of this research are likely to result in a much clearer understanding of the issues you raise.

*I’m unsure about including the paragraph on the expanding role of practice nurses, as you concede this is not an integral part of your research, or indeed a research question. I wouldn’t have thought a full paragraph in this section is warranted. If it is included, perhaps there should be an analysis of the evidence in the literature on the role of nurse vs paramedic in general practices.*

 Thank you for this consideration. We agree that we had not anticipated that the issue of consolidating and expanding the role of the practice nurse would have represented a major theme. None the less this was highlighted again and again by research participants from general practice during the data collection. We do address both the dearth of literature considering practice nursing in Ireland and some literature considering potential role conflict with nursing in the discussion. Beyond this we are not aware of a significant literature that examines the evidence for nurse vs paramedic roles in general practices.

*Strengths and Limitations:*

*I’m unsure what you mean when stating that a strength was that the study used published guidelines.*

 Clarified – ‘These include the use of published reporting guidelines to inform design from the planning stage’

*Unless I missed it, this is the first mention I can see of PPI involvement. Consider inserting it into the methods section too.*

 Thank you - -PPI involvement is detailed in the early part of the Methods section – we have moved this paragraph above rather than below the heading Ethical Approval for clarity.

# Peer review: Reviewer response for version 1

**DOI:** 10.21956/hrbopenres.14787.r32235

**Published:** 2022-06-17

**Authors:** Blandine French

**Affiliations:** 1 Academic Unit of Mental Health & Clinical Neurosciences, Faculty of Medicine & Health Sciences, University of Nottingham, Nottingham, UK

This article examines the role of paramedics in general practice in Ireland. Through stakeholders interviews, it explores the different views of healthcare professionals on the place and role that have paramedics in general practice.

 This study is well conducted and covers an interesting and important topic. The article is well written and clear and the authors did a great job in establishing the background and the importance of the topic. A few points can be raised, mainly around the results and analysis conducted.

 Overall comments: Well written but missing essential punctuation, especially commas throughout. For example, second paragraph of discussion: “For some consideration of paramedic…” the missing comma after some makes this sentence very difficult to understand. Also, the word "highlighted" is excessively used in the result section. It feels very repetitive. In the abstract, result section, spelling mistake at GPs (GPs, not GP’s).

**Introduction:**

- Introduction is very clear and well structured.
- One sentence didn’t make sense to me: “Ninety percent of GPs in Ireland participate in out-of-hours co-operatives”. What are co-operatives?
- “Although GP numbers increased by 20% between 2005 to 2015, there remains significant concern regarding a growing shortage of GPs, which is expected to exceed 1,000 by 2025”. I don’t understand this statement either, could you explain better why if there is a 20% increase, there is a shortage?

**Methods:**

- In data collection, small edit but did you use tapes to record the interview? I assume not and this should read recordings. Full stop missing before last sentence of this paragraph.

**Results:**

- While the article is good, the results for me highlight the most problematic section. The analysis is not done thoroughly enough. The authors did a good job at synthesising the themes, however, they have not gone into enough depth. Too many quotes in proportion to explanation of the themes. Also the explanations are at times just descriptive of the quotes which is not what thematic analyses is about. Finally, the analyses focuses too much on individual findings. On page 7 for instance, the authors state that “one GP highlighted this, then another highlighted that, then another said this, and dedicate a paragraph for each participant. This show the lack of depth in the analysis as the themes should describe overall experiences and synthesise topics rather than focus on individual quotes separately. The quotes are meant to support an important point rather than be an entity on their own.
- But I don’t think much work needs to be done to make this section more representative of good thematic analyses. By combining the points together, taking the analysis a step further, more condensed and taking a lot of quotes off (keeping one/two quote per point), the analysis will be much better.

**A few minor points:**

- Figure one is confusing. After reading the article I understood it but before it didn’t make much sense as we can’t tell which are themes, which are subthemes and what is the main key points/themes of the study.
- The 999 system on page 6 needs to be explained. This is not mentioned earlier and it assumes that the reader knows what it is, which personally I don’t.
- Check the number of times "highlighted" has been used and update.
- “Varied perception of the potential role”. This theme (or subtheme?) is too vague. The role of whom? Paramedics in primary care or vice versa? Please make it more clear.

**Discussion:**

- The discussion is good, clear link to previous studies and rationale. The first paragraph of Comparison with existing literature, the link to how the Booker study maps on to the findings should be made clearer. It is a great point and it would be nice to see exactly how this maps to the findings.

Is the work clearly and accurately presented and does it cite the current literature?

Yes

If applicable, is the statistical analysis and its interpretation appropriate?

Partly

Are all the source data underlying the results available to ensure full reproducibility?

No source data required

Is the study design appropriate and is the work technically sound?

Yes

Are the conclusions drawn adequately supported by the results?

Yes

Are sufficient details of methods and analysis provided to allow replication by others?

Yes

Reviewer Expertise:

Primary care, neurodevelopmental disorders, thematic analyses

I confirm that I have read this submission and believe that I have an appropriate level of expertise to confirm that it is of an acceptable scientific standard, however I have significant reservations, as outlined above.

# Response

**Published:** 2022-07-06

**Authors:** Tomás Barry

We are very grateful for your review. Amendments and responses to your comments are below.

*‘Well written but missing essential punctuation, especially commas throughout. For example, second paragraph of discussion: “For some consideration of paramedic…” the missing comma after some makes this sentence very difficult to understand. Also, the word "highlighted" is excessively used in the result section. It feels very repetitive. In the abstract, result section, spelling mistake at GPs (GPs, not GP’s).’*

 We have amended as advised.

*Introduction: One sentence didn’t make sense to me: “Ninety percent of GPs in Ireland participate in out-of-hours co-operatives”. What are co-operatives?*

 Co-operatives are the general model for out-of-hours GP services in Ireland. For clarity we have replaced this with ‘out-of-hours care’.

*“Although GP numbers increased by 20% between 2005 to 2015, there remains significant concern regarding a growing shortage of GPs, which is expected to exceed 1,000 by 2025”. I don’t understand this statement either, could you explain better why if there is a 20% increase, there is a shortage?*

 Now reads ‘Although GP numbers increased by 20% between 2005 to 2015, there remains significant concern (in the context of population increases and a shift toward care in the community) regarding a growing shortage of GPs, which is expected to exceed 1,000 by 2025’.

*Methods:*

*In data collection, small edit but did you use tapes to record the interview? I assume not and this should read recordings. Full stop missing before last sentence of this paragraph.*

 We have amended as advised.

*Results:*

*While the article is good, the results for me highlight the most problematic section. The analysis is not done thoroughly enough. The authors did a good job at synthesising the themes, however, they have not gone into enough depth. Too many quotes in proportion to explanation of the themes. Also the explanations are at times just descriptive of the quotes which is not what thematic analyses is about. Finally, the analyses focuses too much on individual findings. On page 7 for instance, the authors state that “one GP highlighted this, then another highlighted that, then another said this, and dedicate a paragraph for each participant. This show the lack of depth in the analysis as the themes should describe overall experiences and synthesise topics rather than focus on individual quotes separately. The quotes are meant to support an important point rather than be an entity on their own. But I don’t think much work needs to be done to make this section more representative of good thematic analyses. By combining the points together, taking the analysis a step further, more condensed and taking a lot of quotes off (keeping one/two quote per point), the analysis will be much better.*

 We have considered your feedback and the study data in detail. We have revised the results section to add further depth of analysis. This includes a revised description of the thematic structure including articulations of themes and subthemes as well as a revised Fig 1. We have removed some individual quotes but have retained quotes and relevant commentary where we judged that a salient issue was highlighted, even if only by an individual participant in the context of the development of a theme.

*A few minor points:*

*Figure one is confusing. After reading the article I understood it but before it didn’t make much sense as we can’t tell which are themes, which are subthemes and what is the main key points/themes of the study.*

 Please see above – Fig 1 has been revised. We do not however anticipate that it can be fully understood without reference to the text.

*The 999 system on page 6 needs to be explained. This is not mentioned earlier and it assumes that the reader knows what it is, which personally I don’t.*

 999 has been changed to EMS system which is described in the introduction section ‘Paramedicine in Ireland’.

*Check the number of times "highlighted" has been used and update.*

 Thank you, we have updated in the discussion section as per your advice but retained the use of ‘highlighted’ in the results section. In this context we believe the word accurately describes the nature of the discourse during the interviews.

*“Varied perception of the potential role”. This theme (or subtheme?) is too vague. The role of whom? Paramedics in primary care or vice versa? Please make it more clear.*

 This sub-theme has been revised and is further explained in the text.

*Discussion:*

*The discussion is good, clear link to previous studies and rationale. The first paragraph of Comparison with existing literature, the link to how the Booker study maps on to the findings should be made clearer. It is a great point and it would be nice to see exactly how this maps to the findings.*

 Thank you – we have added a sentence to emphasise participants in our study noted that there was potential for paramedics to provide additional general practice capacity in Ireland across the three overarching models described by Booker and Voss.
